# Physical exposures in the work environment during pregnancy – a challenge for risk assessment

**DOI:** 10.5271/sjweh.4128

**Published:** 2023-11-01

**Authors:** Jenny Selander

**Affiliations:** Head of Unit of Occupational Medicine; Institute of Environmental Medicine; Karolinska Institutet

Pregnant women have been preforming work-related activities during pregnancy since time immemorial, from the traditional hunter-gatherer or forager society to today’s modern world. But ever since our society has been industrialized, exposure patterns for pregnant women have changed dramatically, and they keep changing. This change is due partly to exposure changes overall in the labor market and partly the changes in the gender balance in different occupations.

To some extent, women have been protected from the most hazardous occupations, since these mainly have been held by men (1). But active strategies within Europe to move towards a gender-balanced work force have increased the number of women seeking employment in hazardous work environments. One example is heavy truck and lorry drivers, who are predicted to be 40% female by 2030 (2). Today, the labor participation rate for women is high, with a total of 67% in Europe and 74–80% in Scandinavian countries (3), leading to a workforce that eventually has as many exposed women as men.

A gender-balanced work force across occupations brings diversity and thus benefits to the work site (4). But it also introduces a challenge, especially in exposed blue-collar occupations. So far, much emphasis has been given to chemical and particles exposure. This is a very important and crucial area for the risk assessment for pregnant workers (5, 6) but not the only hazard present in occupational settings. Too little attention has been paid to physical factors in the work environment during pregnancy in association with health effects, even though physical factors are more prevalent than chemical and particles exposure in occupational settings. The exception is physical load, about which several original articles and reviews have been published (7–9). But physical exposure is a broad concept and also includes exposure to temperature, whole-body vibration, and noise.

Among the physical exposures in occupational settings, physically strenuous work has been the most studied. This area includes work postures, heavy lifting, standing/walking, sedentary work as well as a cardiovascular strain from physical labor. Recent reviews show an overall modest effect for physically strenuous work during pregnancy as well as pregnancy complications and adverse birth outcomes (10). In summary, the evidence so far concludes that pregnant workers should avoid occasional heavy lifting and lifting >10 kg in general (9). Heat in residential settings during pregnancy has been extensively studied (11), and some studies have also focused on occupational settings. An association between heat exposure and heat stress in relation to pregnancy complications as well as birth outcomes has been reported (12). But the evidence is not strong enough to recommend specific temperature levels in occupational settings for pregnant workers.

Only a few studies have investigated noise and whole-body vibration and its effect on pregnant workers. In both areas, reviews have shown inconclusive results (13, 14) and, since then, only a handful studies have been published, including only one large scale cohort study divided into five manuscripts (15–19). The evidence so far suggests that working full-time (8 hours) in weighted average of >80dBA occupational noise and >0.5m/s^2^ whole-body vibration is associated with an increased risk of pregnancy complications and negative birth outcomes. But since these findings come from one cohort, they need to be confirmed in other high quality cohort studies where levels of exposure can be assessed.

An important review on the evidence so far regarding physical exposures during pregnancy and preterm birth is on the way; the method and protocols have already been published (20), but a review is only as good as its included studies, and we need more high-quality original studies in this research area. Overall, more prospective cohort studies with objectively assessed exposures are needed to be able to identify a safe level for occupational physical exposures during pregnancy. So far, the current evidence on physical load is predominantly based on self-reported physical load, and future studies should focus on high quality objective exposure assessment to increase the level of evidence within this part of the research field.

To be able to progress, future studies also need detailed information on absence from work. In many countries, absence from work due to pregnancy benefit, sick leave and parental leave is common during pregnancy. In Sweden, 7 out of 10 women apply for leave of absence benefits at some point during pregnancy [21). In contrast to chemical exposures, that can bioaccumulate, physical exposures predominately affect the working mother during pregnancy. Hence, to correctly assess occupational exposure to physical factors during pregnancy in relation to health effects in the mother and child, absence from work needs to be addressed properly. Associations can otherwise be missed due to misclassification of exposure. This have been shown in a large-scale cohort study from Sweden, where associations only were found in full time workers with low leave of absence during pregnancy [16-18).

Leave of absence data is also needed to assess the potential beneficial effect that leave of absence can have on the pregnant worker. This was shown in Skröder et al 2021, where pregnant women highly exposed to whole-body vibration only had an increased risk of preterm birth if they had few days of leave of absence during pregnancy. With increased levels of leave of absence from work, women highly exposed to whole-body vibration at work had the same risk of delivering preterm as the general working population. In high levels of leave of absence data, women highly exposed to whole-body vibration had a lower risk of preterm birth then the general population with the same level of absence from work, indicating a healthy worker effect [16).

Leave of absence during pregnancy can also be seen as an intermediate measure, since early health effects can lead to an increased level of sick leave, parental leave or pregnancy benefit. An association between exposure at the workplace and level of absence from work have been seen in a recent Danish study and in a recent review [22, 23).

High quality environmental epidemiological studies have been of use when assessing the effect of occupational exposure during pregnancy to chemical and particle exposure on pregnancy complications and birth outcomes. But for physical exposures, the exposures and levels differ substantially between environmental and occupational settings and some of the mechanisms. This is true for the mechanism between noise and health, where occupational and residential exposure to noise both are associated with a stress mechanism, but were only the residential exposure contribute to sleep disturbance and related health effects [13).

An exposome approach is needed to identify and assess all the potential risk factors for pregnant women [24). In the exposome concept, multiple exposures in a life course perspective are assessed, with a focus on vulnerable stages, such as pregnancy. It is important to adjust for other co-exposures in the work environment, such as chemical and particle exposure, psychosocial exposures, shift work and other physical exposures when assessing the relationship between occupational exposure to one physical factor and the outcome. These exposures are partly correlated with each other [15-19). Few previous studies have been able to adjust for other occupational exposures at work when investigating individual occupational exposures. Very few studies examined the interaction of occupational exposure and how they jointly contribute to the risk of health effects in the mother and child, even though most of these exposures correlate in the work place [25). Some of the existing birth cohort studies can be regarded as hidden treasures for occupational data, with detailed information on occupational environment of the parents that many times have not been used [26). We need to be able to produce valid exposure-response curves and thereby ensure a safe work environment for the pregnant worker and her child. But also, to avoid excluding women from the labor market during pregnancy unnecessary. An unnecessary exclusion from the labor market can hinder female workers career advancement to the next level of their career and lead to lower salaries, and in the end lower pensions compared to men. So, a well-balance discussion based on high quality evidence can provide a safe and non-discriminatory work environment for pregnant women.

Regarding chemical and particle exposure, an equal low level of exposure for both men and women in reproductive ages can reduce reproductive effects successfully. In physical exposures, there is less need to reduce exposure levels for all, unless these are associated with other health outcomes. It is mainly women of reproductive age who need to be protected, preferably early on since at least whole-body vibration is suspected to be associated with miscarriages. A better system to identify and inform pregnant workers already at the time of their prenatal care registration (usually in gestational week 10) is needed.

Overall, there remains a significant knowledge gap regarding the effect of physical occupational exposures during pregnancy and health effects among children. More high-quality cohort studies with objectively assessed exposure that have access to leave-of-absence-during-pregnancy data are needed to increase the level of knowledge in this important area so researchers can generate accurate exposure–response functions and provide correct and well-balanced advice to occupational health services, employers, and pregnant workers.
